# Effect of different doses of aspirin on the prognosis of Kawasaki disease

**DOI:** 10.1186/s12969-020-00432-x

**Published:** 2020-06-11

**Authors:** Jinxin Wang, Huiqiao Chen, Hongying Shi, Xuting Zhang, Yiping Shao, Biyao Hang, Zhipeng Xu, Xing Rong, Maoping Chu, Huixian Qiu

**Affiliations:** 1 Children’s Heart Center, The Second Affiliated Hospital and Yuying Children’s Hospital, Institute of Cardiovascular Development and Translational Medicine, Wenzhou Medical University, Wenzhou, 325027 Zhejiang China; 2 Department of Preventive Medicine, School of Enviromental Science and Public Health, WenZhou Medical University, Wenzhou, 325035 Zhejiang Province People’s Republic of China

**Keywords:** Aspirin, Kawasaki disease, Dose, Prognosis, Coronary artery damage, Intravenous immunoglobulin

## Abstract

**Background:**

Kawasaki disease (KD) is the leading cause of acquired heart disease in children, and is steadily increasing in prevalence in East Asia. KD is often complicated by coronary artery damage, including dilatation and/or aneurysms. Aspirin is used with intravenous immunoglobulin (IVIG) to prevent coronary artery abnormalities in KD. However, the role and optimal dose of aspirin remain controversial. Identifying the dose of aspirin in the acute phase will facilitate development of a more appropriate treatment strategy in improving the outcome of KD.

**Methods:**

A total of 2369 patients with KD were retrospectively analyzed and divided into three groups according to the aspirin dose: 510 in group 1 (20–29 mg/kg/day), 1487 in group 2 (30–39 mg/kg/day), and 372 in group 3 (40–50 mg/kg/day). The differences in laboratory data, rate of IVIG resistance and coronary artery damage were compared among the groups.

**Results:**

There was no difference in the incidence of coronary artery aneurysms (CAAs) in group 1 compared with groups 2 and 3 (2 weeks of illness: 2.94% vs. 1.90% vs. 3.36%; 3–4 weeks of illness: 1.94% vs. 2.32% vs. 2.65%). The risk for developing CAA was not reduced at 2 weeks of illness onset in groups 2 and 3 compared with group 1 (adjusted OR = 1.05, 95% confidence interval: 0.34–3.18; aOR = 1.81, 95% CI: 0.42–7.83). Furthermore, the risk for developing CAA was not reduced at 3–4 weeks of illness onset in groups 2 and 3 (aOR = 2.63, 95% CI: 0.61–11.28; aOR = 0.52, 95% CI: 0.03–9.54). There was no significant difference in the rate of IVIG resistance among the groups. Platelet levels after IVIG treatment in group 1 were significantly lower than those in groups 2 and 3 (522.29 × 10^9^/L, 544.69 × 10^9^/L, and 557.77 × 10^9^/L, *p* = 0.013). C reactive protein of the 30-40 mg/kg*day group was slightly higher than the other two groups. (7.76, 8.00, and 7.01 mg/L, *p* = 0.028).

**Conclusions:**

Aspirin at the dose of 20–29 mg/kg/day dose not increase the risk of coronary artery damage and IVIG resistance compared with the dose of 30–50 mg/kg/day. This low dose may have a lower risk for a potential effect on liver function.

## Background

Kawasaki disease (KD) is a vascular inflammatory disease. KD is currently the most common acquired heart disease, and has already surpassed the rate of rheumatic heart disease [1]. The incidence of KD in Japan and South Korea is the highest in the world, with an incidence of 330 and 194.7 per 100,000 people, respectively. However, the incidence of KD in the United States, the United Kingdom, and Australia ranges from 8.39 to 25 per 100,000 people [2–6]. KD primarily endangers children and can cause coronary artery aneurysms (CAAs). The incidence of CAA has fallen sharply after standardized treatment [7].

The initial treatment of KD with IVIG has generally reached consensus worldwide. However, there is still controversy about the appropriate aspirin dose to be used as a secondary cornerstone treatment. In North America, under the guidance of the America Heart Association (AHA) guidelines, high-dose aspirin (80–100 mg/kg/day) is used in the acute phase of KD [7]. In Asian regions under the guidance of the Japanese guidelines, moderate-dose aspirin (30–50 mg/kg/day) is widely used [8], and the aspirin dose in Western Europe is the same as that in Asia. Some Centers are opting to use low dose aspirin (3–5 mg/kg/day) [9].

Because of a lack of high-quality, randomized, and controlled trials, each dose has its supporting basis and cannot be completely unified. To balance the risks and benefits of aspirin, we performed a study to determine whether different doses of aspirin affect the outcome of KD including IVIG resistance and coronary artery damage.

## Materials and methods

### Subjects

We reviewed the data of children who were diagnosed with KD at the Second Affiliated Hospital and Yuying Children’s Hospital of Wenzhou Medical University from January 2005 to December 2018. There were initially a total of 2768 children. We included children who were treated with 20–50 mg/kg/day of aspirin in the study. A total of 42 children who did not use aspirin and 301 children who received less than 20 mg/kg/day or more than 50 mg/kg/day were excluded. At the same time, we excluded seven patients without cardiac ultrasound results and 49 in whom IVIG use was unknown. Finally, 2369 children were included in this study. The study flowchart is shown supplementary Fig. 1. All children were divided into the following groups according to the aspirin dose: group 1 (20–29 mg/kg/day), group 2 (30–39 mg/kg/day), and group 3 (40–50 mg/kg/day).

### Data collection

We collected the following data from the electronic medical record: weight, age (months), gender, time of IVIG treatment (the number of days that patients treated with IVIG after the onset of disease), and the regime of IVIG treatment. Laboratory data before and after IVIG treatment were recorded, including the white blood cell count (WBC), absolute neutrophil count (ANC), levels of hemoglobin (Hb), alanine aminotransferase (ALT), aspartate aminotransferase (AST), C-reactive protein (CRP), albumin, fibrinogen (FIB), and D-dimer, and the erythrocyte sedimentation rate (ESR). Echocardiogram examinations were performed at 2 and 3–4 weeks after onset of illness.

### Criteria for diagnosis of KD

The diagnosis of KD was based on the 2017 AHA guidelines [7] as follows: (1) fever ≥5 days, (2) eye conjunctival hyperemia, (3) oral changes, (4) polymorphic rash, (5) hand and foot sclerosis or peeling, and (6) cervical lymphadenopathy.

### Resistance to IVIG

IVIG resistance was defined as when body temperature did not fall to normal 48 h after the end of IVIG infusion, or increased again above 38.0 °C [10].

### Coronary artery damage

CAA was defined as a coronary artery diameter ≥ 4 mm [11].

### Statistical analysis

Statistical analysis were performed by IBM SPSS (version 23). Continuous variables are shown by the mean ± standard deviation or median and interquartile range. Categorical variables are expressed by numbers and percentages. The chi-square test was used to compare categorical variables. The associations between aspirin doses and coronary artery damage or IVIG resistance were analyzed using multiple logistic regression. A *p* value of < 0.05 was considered statistically significant.

## Results

### Demographic characteristics and IVIG treatment options

Of 2369 children diagnosed with KD in the study, 510 received 20–29 mg/kg/day of aspirin treatment, 1487 received 30–39 mg/kg/day, and 372 received 40–50 mg/kg/day. There were significant differences in mean weight and age between the three groups (both *p* < 0.001). There were also significant differences in the proportion of no use of IVIG among the three groups, among which group 1 had the highest proportion, followed by group 2 and then group 3 (5.29% vs 3.09, 1.34%, *p* < 0.001). No significant difference was found with respect to treatment time of IVIG and gender among the three groups (Table 1).

**Table 1:** Demographic characteristics

Group	1	2	3	*P*-value
N	510	1487	372	
Weight (kg)	11.50 (10.50–15.45)	11.30 (9.00–14.00)	10.50 (7.50–13.00)	**<0.001**
Age (month)	22.14 (14.83–39.93)	19.43 (10.66–35.14)	15.79 (6.45–29.30)	**<0.001**
IVIG treat time (day)	6.00 (6.00–8.00)	6.00 (6.00–7.00)	6.00 (6.00–7.00)	0.574
SEX				0.574
Male	337 (66.08%)	948 (63.75%)	244 (65.59%)	
Female	173 (33.92%)	539 (36.25%)	128 (34.41%)	
IVIG treat option				**0.004**
No use	27 (5.29%)	46 (3.09%)	5 (1.34%)	
1 g or 2 g options	483 (94.71%)	1441 (96.91%)	367 (98.66%)	

Data are shown as number (N), mean (SD), or median (Q1–Q3)/N (%)

IVIG: intravenous immunoglobulin

Group 1: patients with Kawasaki disease treated with aspirin at 20–30 mg/kg/d in the acute stage

Group 2: patients with Kawasaki disease treated with aspirin at 30–40 mg/kg/d in the acute stage

Group 3: patients with Kawasaki disease treated with aspirin at 40–50 mg/kg/d in the acute stage

### Laboratory tests before IVIG therapy

Hb levels in group 1 were significantly higher than those in the other two groups (*p* < 0.001, Table 2). There were no significant differences in the values of the WBC, PLT, ANC, CRP, and the ESR. A liver function test showed that albumin levels were highest in group 1 and lowest in group 3 (p < 0.001). ALT and AST levels were not significantly different among the three groups. For coagulation measurements, except for FIB levels, which were significantly different among the three groups (*p* = 0.002), the Prothrombin time (PT), Activated partial thromboplastin time (APTT), Thrombin time (TT), and D-dimer levels were not significantly different among the three groups.

**Table 2:** Laboratory tests before aspirin treatment

Group	1	2	3	*P*-value
N	510	1487	372	
WBC1(10^9/L)	15.15 (11.91–19.37)	15.57 (12.24–19.80)	15.42 (12.70–19.86)	0.276
HB1(g/L)	111.64 ± 11.34	110.42 ± 11.34	108.48 ± 11.05	**<0.001**
PLT1(10^9/L)	379.24 ± 135.27	388.75 ± 136.16	388.44 ± 143.93	0.392
ANC1(10^9/L)	10.79 ± 5.15	10.76 ± 4.99	10.64 ± 4.80	0.903
CRP1(mg/L)	70.10 (35.00–113.87)	75.00 (42.49–118.01)	81.40 (47.55–117.00)	0.196
ESR1(mm/h)	37.50 ± 17.14	36.99 ± 16.90	35.84 ± 17.56	0.412
ALT1(u/L)	35.00 (17.00–91.00)	34.00 (18.00–92.50)	33.00 (19.00–75.00)	0.552
AST1(u/L)	33.50 (25.00–50.25)	31.50 (24.00–51.00)	29.00 (24.00–52.00)	0.225
Albumin1(g/L)	36.52 ± 6.26	36.19 ± 6.28	34.63 ± 6.58	**<0.001**
PT1(s)	13.82 ± 0.99	13.82 ± 1.05	13.97 ± 1.63	0.165
APTT1(s)	43.34 ± 5.94	43.71 ± 6.71	43.11 ± 5.99	0.331
FIB1(g/L)	1.23 (0.85–2.16)	1.43 (0.87–2.16)	1.37 (0.82–2.28)	**0.002**
TT1(s)	14.84 ± 0.90	14.89 ± 1.23	14.87 ± 1.09	0.804
D-Dimers1 (ug/ml)	1.23 (0.85–2.16)	1.43 (0.87–2.16)	1.37 (0.82–2.28)	0.328

Continuous data are presented as mean ± SD if data are normally distributed and expressed as median (interquartile range) if data are not normally distributed

*WBC* white blood cell count, *ANC* absolute neutrophil count, *CRP* C reactive protein, *ESR* erythrocyte sedimentation rate, *ALT* alanine aminotransferase, *AST* aspartate aminotransferase, *PT* prothrombin time, *APTT* activated partial thromboplastin time, *FIB* fibrinogen, *TT* thrombin time, *Hb* hemoglobin, *PLT* platelet Count

### Laboratory tests after IVIG therapy

Patients with KD had higher Hb levels in group 1, followed by group 2 and then group 3 (*p* < 0.001, Table 3). PLT levels in group 1 were significantly lower than those in groups 2 and 3 (*p* = 0.013). CRP levels were significantly different among the three groups (*p* = 0.028). Albumin levels were significantly higher in group 1 compared with the other two groups (*p* < 0.001). Similar to albumin levels, FIB levels in group 1 were higher compared with those in the other two groups (p < 0.001). However, ALT, AST, and D-dimer levels, and the PT, APTT, and TT were not significantly different among the three groups.

**Table 3:** Laboratory tests after aspirin treatment

Group	1	2	3	*P*-value
N	510	1487	372	
WBC2(10^9/L)	8.37 ± 3.39	8.67 ± 3.36	8.94 ± 3.90	0.065
HB2(g/L)	109.80 ± 11.34	108.46 ± 10.55	106.32 ± 10.71	**<0.001**
PLT2(10^9/L)	522.29 ± 173.58	544.69 ± 172.46	557.77 ± 193.50	**0.013**
ANC2(10^9/L)	2.44 (1.68–3.93)	2.60 (1.66–3.93)	2.45 (1.56–3.76)	0.416
CRP2(mg/L)	7.76 (3.00–14.42)	8.00 (3.83–15.18)	7.01 (3.00–14.28)	**0.028**
ESR2(mm/h)	35.57 ± 19.49	35.82 ± 19.64	32.48 ± 16.86	0.439
ALT2(u/L)	27.00 (18.00–43.00)	28.00 (19.00–44.00)	27.00 (18.00–46.75)	0.606
AST2(u/L)	42.00 (35.00–55.00)	42.00 (35.00–55.00)	43.00 (35.00–61.00)	0.456
Albumin2(g/L)	36.01 ± 4.30	35.40 ± 4.74	34.40 ± 4.55	**< 0.001**
PT2(s)	12.82 ± 0.91	12.81 ± 0.78	12.81 ± 0.69	0.978
APTT2(s)	39.51 ± 6.13	39.69 ± 5.67	38.92 ± 5.26	0.251
FIB2(g/L)	4.19 ± 0.92	4.09 ± 0.94	3.86 ± 1.00	**< 0.001**
TT2(s)	17.38 ± 1.41	17.41 ± 1.17	17.34 ± 1.24	0.780
D-Dimers2 (ug/ml)	0.98 (0.71–1.49)	0.92 (0.68–1.42)	0.88 (0.62–1.47)	0.198

Continuous data are presented as mean ± SD if data are normally distributed and expressed as median (interquartile range) if data are not normally distributed

*WBC* white blood cell count, *ANC* absolute neutrophil count, *CRP* C reactive protein, *ESR* erythrocyte sedimentation rate, *ALT* alanine aminotransferase, *AST* aspartate aminotransferase, *PT* prothrombin time, *APTT* activated partial thromboplastin time, *FIB* fibrinogen, *TT* thrombin time, *Hb* hemoglobin, *PLT* platelet Count

### CAA and IVIG resistance

The proportion of IVIG resistance was not significantly different among the three groups (Table 4). Additionally, the incidence of CAA was not different among the three groups at 2 weeks and 3–4 weeks after onset of illness.

**Table 4:** Outcome

Group	1	2	3	*P*-value
IVIG-unresponse	118 (24.48%)	344 (23.89%)	98 (26.70%)	0.534
CAA(2 weeks)	14 (2.94%)	27 (1.90%)	12 (3.36%)	0.147
CAA(3–4 weeks)	7 (1.94%)	26 (2.32%)	7 (2.65%)	0.843

*CAA* coronary artery aneurysm, *IVIG* intravenous immunoglobulin

### Effect of aspirin on CAA and IVIG resistance

Multiple logistic regression was used to analyze the relationship between the dose of aspirin and the risk of CAA or IVIG resistance after adjusting for age, weight, sex, IVIG treatment options, IVIG treatment time, Hb levels, albumin levels, and FIB levels (Table 5). Intriguingly, there was no significant correlation between the dose of aspirin and the prevalence of CAA at 2 weeks or 3–4 weeks after onset of illness, even after adjusting for the factors of age, weight, sex, IVIG treatment options, IVIG treatment time, Hb levels, albumin levels, and FIB levels before aspirin treatment. Two weeks after the onset of illness, treatment with 30–339 mg/kg/day of aspirin was associated with an odds ratio of 1.05 for CAA (95% confidence interval [CI]: 0.34–33.18, *p* = 0.9352), whereas 40–350 mg/kg/day was associated with an odds ratio of 1.81 (95% CI: 0.42–37.83, *p* = 0.4273). The risk difference of CAA at 3–34 weeks after onset of illness adjusted for potential confounders was 2.63 (95% CI: 0.61–11.28, *p* = 0.1921) in group 2 and 0.52 (95% CI: 0.03–9.54, *p* = 0.6589) in group 3. Analysis that was non-adjusted or adjusted for the same related factors also showed no correlation between the risk of IVIG resistance and doses of aspirin combination therapy.

**Table 5:** Multiple regression analyses

	Non-adjusted	Adjusted
IVIG-unresponse		
Group 1	1.0	1.0
Group 2	0.97 (0.76, 1.23) 0.7922	1.07 (0.77, 1.49) 0.6958
Group 3	1.12 (0.82, 1.53) 0.4617	1.17 (0.75, 1.85) 0.4895
CAA(2 weeks)		
Group 1	1.0	1.0
Group 2	0.64 (0.33, 1.23) 0.1840	1.05 (0.34, 3.18) 0.9352
Group 3	1.15 (0.53, 2.52) 0.7261	1.81 (0.42, 7.83) 0.4273
CAA(3-4 weeks)		
Group 1	1.0	1.0
Group 2	1.20 (0.51, 2.78) 0.6785	2.63 (0.61, 11.28) 0.1921
Group 3	1.37 (0.48, 3.96) 0.5572	0.52 (0.03, 9.54) 0.6589

Data are shown as OR (95% CI) p value

*CAA* Coronary artery aneurysm, *IVIG* intravenous immunoglobulin

The model was adjusted for age, weight, sex; IVIG treatment options, IVIG treatment time, Hb levels, albumin levels, and FIB levels

## Discussion

Application of aspirin in KD was reported earlier than that of IVIG [12]. Previous studies [13] have suggested that high-dose aspirin combined with IVIG is beneficial for preventing coronary damage. Subsequent studies have indicated that coronary damage is only negatively correlated with the dose of IVIG, but not associated with aspirin [14]. However, to date, Japanese [15] and American [7] guidelines still recommend the use of moderate-dose aspirin (30–0 mg/kg/day) and high-dose aspirin (80–100 mg/kg/day), respectively. Clinically, because of the pharmaceutical dosage form, tablet aspirin is usually 50 mg, 100 mg, but the weight of the children varies. Clinical use of guidelines recommend 30–50 mg/kg of body weight, but the actual accurate calculation may be 20–50 mg. Coupled with the actual practice habits of different centers, some centers rapidly reduce the dose of aspirin to 3–5 mg/kg/day after being afebrile, and even some patients with atypical KD without fever before IVIG use usually have a low dose (3–5 mg/kg/day). Therefore, we took into account the case of a dose > 20 mg/kg/day in the acute phase of KD. In the present study, no significant difference was found in gender and IVIG treatment time among the groups. The difference found in age is related to the true clinical situation in the case of low-weight children and aspirin dosage forms. With a younger age and lower weight, the relative value of aspirin/weight is greater. At the same time, in younger children, clinicians are more careful and the rate of IVIG use is higher.

In recent years, many studies have been performed to determine the optimum dose of aspirin, and they have shown that the current recommended dose is not reasonable. Ogata et al. reported that there was no significant difference between moderate and high aspirin dose in KD [16]. A multicenter, randomized, single blind trial in Taiwan also showed that there was no significant difference in short-term coronary lesions of KD between high-dose and low-dose aspirin in the acute phase [17]. Huang et al. [18] reported that low-dose aspirin and moderate-dose aspirin had no effect on coronary artery damage in KD, although they did not show cardiac B-ultrasound time. Recent reports by Brooks et al. And Zheng et al. demonstrate that low-dose aspirin has there was no no significant difference in fever days and coronary artery damage rate between low-dose and traditional high-dose aspirin dose groups [19, 20]. This finding suggested that increasing the aspirin dose in the acute phase of KD may not needed. Furthermore, Migally et al. [21] found that the duration of high-dose aspirin had no relationship with long-term prognosis of the coronary artery. This evidence suggests that high-, moderate-, and low-dose aspirin appear to have no significant difference in the prognosis of KD. Pi et al. [22] reported that 11-dehydrothromboxane B2, the final product of the thromboxaneA2 pathway, was not reduced by using aspirin. Additionally, conflicting conclusions were reached by clinical researchers who showed that administration of different doses of aspirin was associated with protection against CAA. Dhanrajani et al. [23] found that IVIG resistance easily occurs with low-dose aspirin. Kim et al. [24] reported that moderate–high-dose aspirin was a risk factor for coronary artery CAA after adjusting for various factors compared with low-dose aspirin. In the present study, our findings showed that there was no significant differences in the incidence of CAA and the proportion of IVIG resistance between KD children who received different doses of aspirin. Our logistic analysis indicated that after adjusting for age, weight, sex, IVIG treatment options, IVIG treatment time, Hb levels, albumin levels, and FIB levels, the risk of CAA was not different among the three groups, indicating that there was no significant correlation between the dose of aspirin and the prevalence of CAA.

As a classic non-steroidal anti-inflammatory drug with acetylsalicylic acid, clinically, aspirin is widely used in preventing cardiovascular disease and postoperative thrombosis. Remarkably, aspirin combines anti-inflammatory (high dose) effects and anti-platelet (low dose) effects. Aspirin inhibits prostaglandin synthetase, reducing prostaglandin production to achieve anti-inflammatory and analgesic effects [25]. The anti-inflammatory effect of aspirin increases with dose accumulation. CRP levels proportionally reflect the grade of inflammation. In the present study, CRP levels were comparable among the groups. But the CRP levels of Group 3 (40–50 mg/kg/day) were lower than those of the other two groups. This finding indicates that relatively high doses of aspirin have better anti-inflammatory effects, although after treatment, CRP levels in the three groups all fell to within the normal range. Aspirin inhibits the Cyclooxygenase pathway and disrupts production of thromboxane thromboxaneA2, thereby reducing platelet aggregation and formation of a thrombus [26]. In the present study, significant differences were also found in PLT levels among three goups, the PLT count after treatment of 20–29 mg/kg/day of aspirin was the lowest among the groups, and to some extent, the relatively low dose of aspirin achieved an antiplatelet effect. Additionally, in the human body, aspirin is mainly absorbed in the stomach and small intestine, and rapidly hydrolyzed to salicylic acid. The rate of aspirin binding to plasma albumin is approximately 80–90%. However, during the acute phase of KD, the inflammatory responses of the gastrointestinal tract and a decrease in serum albumin levels lead to decreased efficacy of aspirin. After inflammation is relieved, gastrointestinal function and albumin levels are rapidly restored, which may even lead to drug toxicity [27]. Our results showed that Hb and albumin levels were significantly higher, and FIB levels were significantly lower in group 1 than those in the other two groups before treatment, while Hb and albumin levels in the three groups after treatment were the same as those before treatment. This finding suggested that the aspirin dose had little effect on these variables. While equally effective in therapeutic benefits, low-dose aspirin produces fewer side effects compared with traditional relatively high-dose aspirin.

Limitations of this study include its single-center and retrospective nature that is prone to indication bias, as well as a loss of follow-up data. However, our study reflects the real clinical condition. Additionally, the Z value could not be used to calibrate coronary damage results because of a lack of height data of children with KD. Therefore, a larger and more in-depth study is required and Z values should be used as possible variations. To the best of our knowledge, no prospective study has been performed to minimize moderate-dose aspirin for preventing CAA in KD.

In conclusion, our data show that in clinical practice, the commonly recommended dosage of 30–50 mg/kg/day of aspirin is not better than 20–29 mg/kg/day.

## Supplementary information

**Additional file 1: Figure 1.** Study flow diagram.

## Data Availability

The datasets used and/or analysed during the current study are available from the corresponding author on reasonable request.

## Abbreviations

KD: Kawasaki disease
IVIG: intravenous immunoglobulin
CAAs: coronary artery aneurysms
CRP: C reactive protein
WBC: white blood cell count
ANC: absolute neutrophil count
Hb: hemoglobin
ALT: alanine aminotransferase
AST: aspartate aminotransferase
ESR: erythrocyte sedimentation rate
PT: prothrombin time
APTT: activated partial thromboplastin time
TT: thrombin time
FIB: fibrinogen
PLT: platelet Count
