# Supplementary figures and images for: Effect of different doses of aspirin on the prognosis of Kawasaki disease
Source: Pediatr Rheumatol Online J. 2020 Jun 11;18:48. doi: 10.1186/s12969-020-00432-x (PMC7291457; doi:10.1186/s12969-020-00432-x)

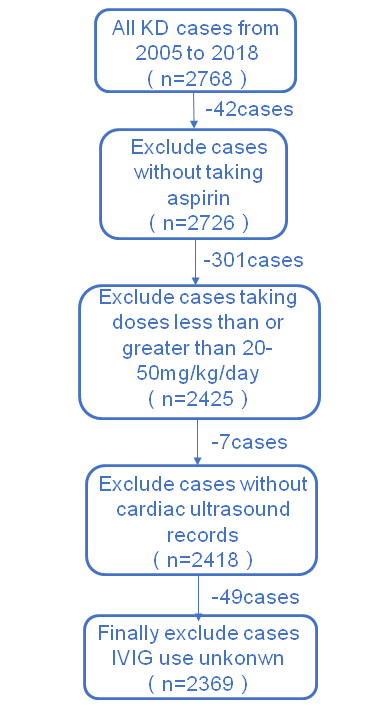

Supplement: Supplementary file 1 — Additional file 1: Figure 1. Study flow diagram. [file 12969_2020_432_MOESM1_ESM.tif]
